# Buschke-Ollendorff Syndrome: A Rare Cause of Unilateral Genu Valgum

**DOI:** 10.7759/cureus.38074

**Published:** 2023-04-24

**Authors:** Şafak Aydın Şimşek, Tolgahan Cengiz, Oğuzhan Muslu, Bedirhan Albayrak, İsmail Büyükceran, Hüseyin Sina Coşkun, Nevzat Dabak

**Affiliations:** 1 Department of Orthopaedics and Traumatology, Ondokuz Mayıs University, Faculty of Medicine, Samsun, TUR; 2 Department of Orthopaedics and Traumatology, Hatay Training and Research Hospital, Hatay, TUR

**Keywords:** genetic skin disease, heterotopic ossicication, buschke-ollendorff, melorheostosis, cortical bone

## Abstract

Buschke-Ollendorff syndrome is a rare, often benign, autosomal dominant skin disorder. This syndrome commonly presents with non-tender connective tissue nevi and sclerotic bony lesions. Characteristic skeletal findings such as melorheostosis and hyperostosis are usually present. Most cases are detected incidentally. Skin lesions appear first and become less noticeable with age. Bone lesions occur in the later decades of life. Another rarely associated symptom, melorheostosis, is manifested by the appearance of wax running through the cortex of the bone. Plain radiographs usually show cortical hyperostosis. This study aims to present a case report of Buschke-Ollendorff syndrome from an orthopedic aspect and emphasize the importance of the disease since it can be easily assessed as a bone tumor. Second, to the best of our knowledge, this is the first case presented with a unilateral genu valgum deformity with a long-term follow-up in the relevant literature.

## Introduction

Melorheostosis is an uncommon skeletal dysplasia that more often involves the lower extremities and may be accompanied by pathologies of mesenchymal origin. It was first described by Leri and Joanny in 1922 [1]. The radiological appearance may be confused with metabolic bone diseases such as osteopoikilosis and osteopathia striata. There is cortical hyperostosis on plain radiographs. This appearance is likened to the flow of melted wax droplets on the edge of a burning candle. Increased activity is determined in the lesion region by bone scintigraphy [2]. In imaging studies, it can be easily confused with bone tumors. The disease may also cause limb-length discrepancies and secondary skeletal deformities due to melorheostosis.

This case report presents a patient with Buschke-Olendorff syndrome, an autosomal skin disorder accompanied by melorheostosis, which is an uncommon skeletal dysplasia causing unilateral genu valgum deformity.

## Case presentation

**Figure 1:**
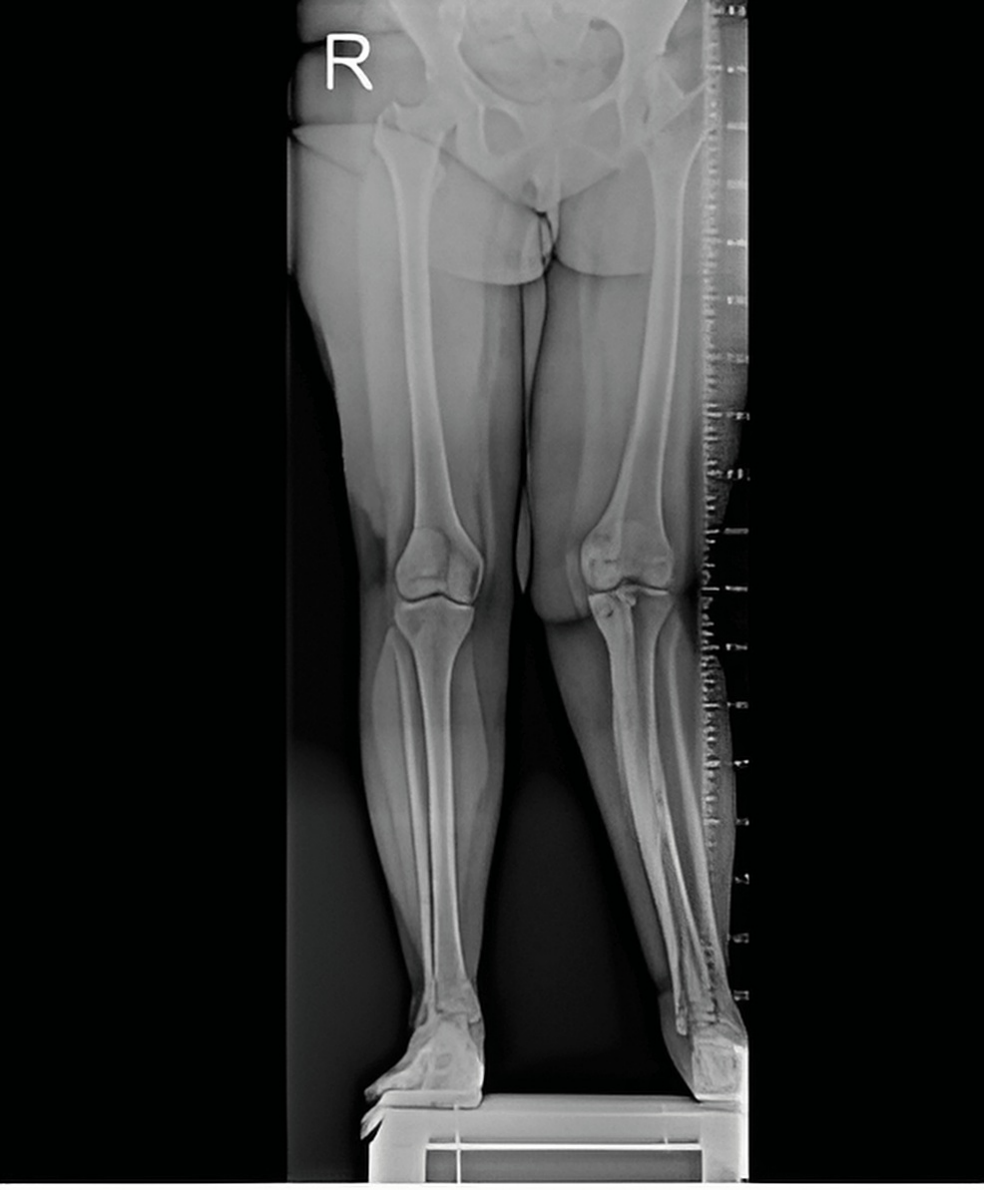
The orthoroentgenogram of the patient with valgus deformity in the left lower limb.

A 19-year-old female patient presented to the orthopedic outpatient clinic with a complaint of pain in the left knee. From her past medical history, it has been learned that she was treated surgically and diagnosed with developmental dysplasia of the left hip during her childhood. Her neurovascular examination was normal, and there was no limb-length inequality. Her pain symptoms were of insidious onset. Also, on the physical examination, the left tibia had a valgus angulation deformity. Skin lesions were observed as well. She had an additional complaint of a palpable mass in the posterior aspect of the left knee, and an orthoroentgenogram was taken (Figure 1).

**Figure 2:**
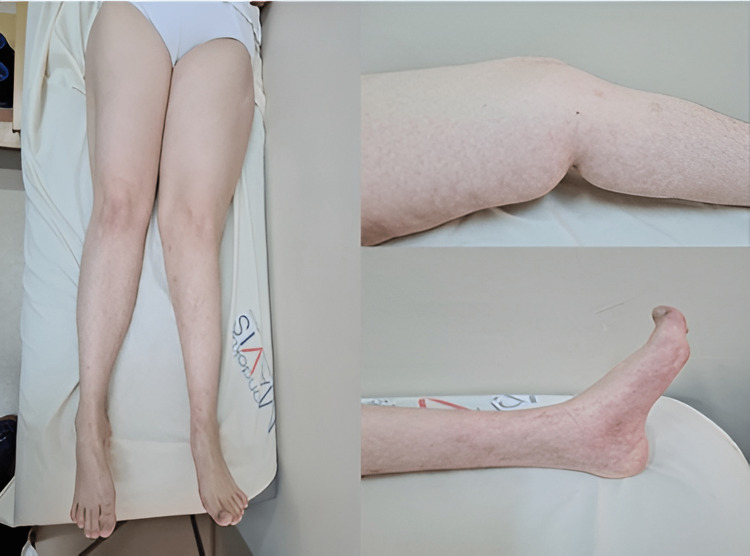
Reticular skin lesions on the medial surface of the left lower extremity.

In further physical examination, the patient had flexion contracture in the left knee, and skin lesions were observed with a reticular pattern over the medial part of the left lower extremity (Figure 2). It was learned that these lesions had developed since birth and were permanent. The patient was referred to the dermatology department, and the pathology result of the skin biopsy supported the diagnosis of elastoma.

**Figure 3:**
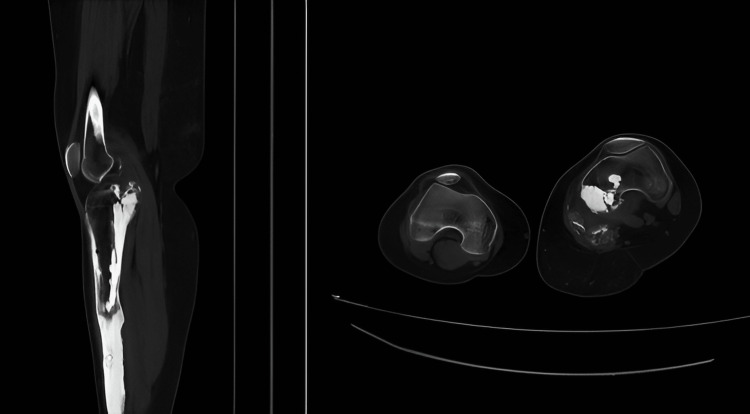
The flow of melted wax appearance - melorheostosis - around the left knee on axial and sagittal sections of the CT scan.

Magnetic resonance imaging (MRI) was performed for the lesion in the left knee, and scintigraphy was carried out for the bone lesions in the left lower extremity. On scintigraphy, an increase was reported in heterogenous osteoblastic activity in the left lower extremity. A computed tomography (CT) examination showed that the bone lesions in the left knee and tibia were consistent with heterogenous ossification (Figure 3).

**Figure 4:**
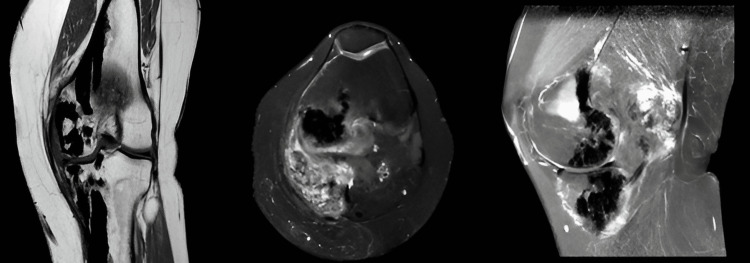
Left knee MR sections showing an area of heterotopic ossification containing calcifications extending from the posterior of the femur medial condyle as far as the adjacent joint capsule. Calcified tubular appearances around the left knee are shown on the coronal plane.

**Figure 5:**
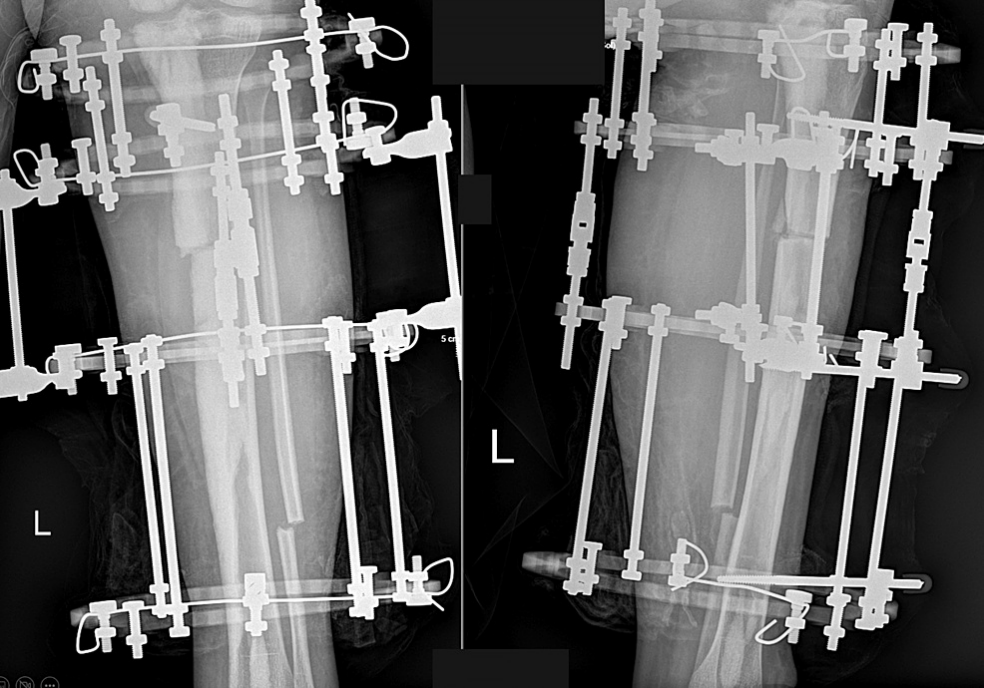
Left tibia anterior-posterior and lateral plain radiograph after the surgical intervention. As seen in the plain radiographs, deformity correction is obtained via the circular frame of Ilizarov.

**Figure 6:**
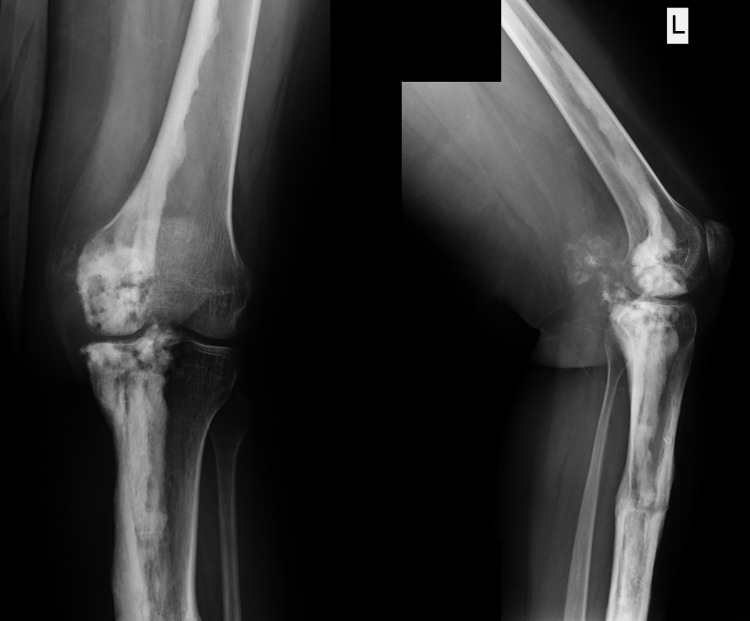
Plain radiographs of the left knee at 3-year follow-up. Although the deformity is corrected, the melorheostosis is still present.

**Figure 7:**
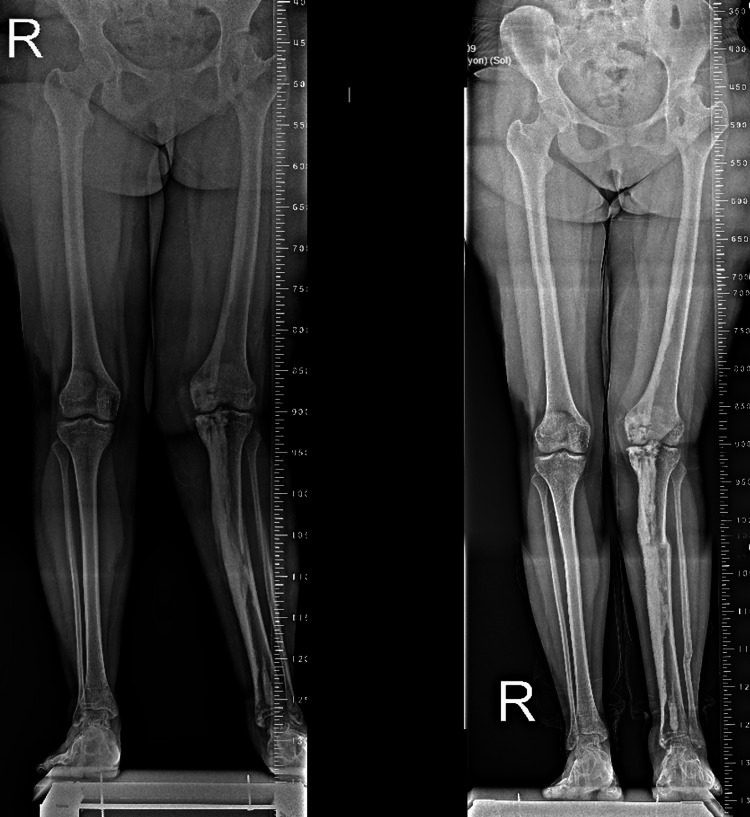
Comparative orthoroentgenograms of the patient. Preoperative and postoperative imaging.

The left knee MR images (Figure 4) were consistent with heterotopic ossification, and the pathology report after the biopsy supported the diagnosis. The multidisciplinary Orthopaedics and Traumatology Bone and Soft Tissue Tumor Council evaluated the patient's clinical findings and imaging results. The patient had no complaints other than palpable swelling, so conservative treatment was chosen for the bone lesions, but Ilizarov's circular external frame is planned to correct the valgus deformity (Figure 5). Plain radiography was used to evaluate the bony structures during the patient's follow-ups. MRI was used to monitor the area of heterotopic ossification in the popliteal region. No significant increase in size or morphological differences was observed in control plain radiographs or MRIs. Final follow-up X-rays are given in Figure 6. The deformity is corrected with slight valgus, but the patient's clinical functions are quite good. Preoperative and postoperative comparative orthoroentgenograms are given in Figure 7.

## Discussion

Buschke-Olendorff syndrome is an uncommon, generally benign, autosomal skin disorder. It emerges with connective tissue nevi and sclerotic bone lesions. Characteristic bone findings may also be present. If the patient's genetic status is unknown, bone findings may be misdiagnosed as malignant cancers [1]. First, skin lesions emerge in the patient, becoming less differentiated with aging. Melorheostosis typically starts insidiously and shows slow progression over time. This disorder can be diagnosed at any age but is generally seen during childhood or adolescence. Almost half of all cases are diagnosed before the age of 20. Bone lesions progress rapidly in childhood, and adult progression is variable [3].

A change may be seen in the bone and muscle structures adjacent to the affected bone in patients with melorheostosis. Young et al. reported thickening in the skin of patients with a skin disorder; the skin in some patients was stretched, shiny, and erythematous, and varices could even be seen in some cases [4].

Bone lesions emerge in the later decades of life. Another uncommon related symptom is the manifestation of melorheostosis with the appearance of melting candle wax along the cortex and cortical hyperostosis seen on radiographs. The localization of extended hyperostosis is the disease's most important radiographic diagnostic feature. In children, lesions may also be seen in striations in the long bones, staining in the pelvis, and small bones such as those in the hands and feet [5].

In addition to the typical radiological findings of melorheostosis, Freyschmidt described three more patterns. The first of these is an osteoma-like lesion in the long axis of the relevant bone, which should be ≥5 cm, include more than one bone, and have eccentric localization. The second pattern is unilateral localization close to the inner surface of the cortex in two or more bones, showing long, dense hyperostotic lines. The third pattern is periarticular lesions similar to myositis ossificans in two or more regions with or without intraosseous hyperostosis. Compared with myositis ossificans, the ossifications should be in a nodular arrangement and not be seen in the lamellar bone structure. In addition, the patient should not have a history of trauma localized in this region [6]. In the current case, the patient had long-standing complaints of swelling in the popliteal region of the left lower extremity. On the MRI examinations, there was an area of heterotopic ossification with calcifications adjacent to the posterior of the medial femoral condyle. The patient also had no history of localized trauma, and no neurological deficit was determined.

Several methods are used to treat pain and deformity related to melorheostosis, including conservative and surgical procedures. The conservative treatments include oral drugs such as biphosphonates, non-steroid anti-inflammatory drugs, and nifedipine. Physical therapy, serial plaster casting, nerve blockage, and sympathectomy are other non-surgical treatment methods. The surgical procedures used in treating patients with melorheostosis are tendon lengthening, extremity lengthening, fibrous tissue excision, fasciotomy, capsulotomy, osteotomy, osteotomy, hyperostosis excision, arthrodesis, contralateral epiphysiodesis, and amputation [3,7]. There is no consensus on the treatment of this rare disease.

In pediatric patients, the parents must know that there could be a postoperative recurrence and contracture in the natural course of the disease. Deformities often recur as fibrotic tissues have not stretched or matched the growing extremity. To correct the deformity caused by melorheostosis, rather than a tendon lengthening procedure alone after completing the bone procedures, it is recommended that contractures be corrected with radical loosening using a wide capsulotomy and tenotomy [4,8]. But even with these radical procedures, recurrence must always be considered.

Genu valgum is a common and familiar disorder in orthopedic outpatient clinics. The causes of the disease can be both physiological and pathological. The pathological entities will worsen over time, while the physiological entities do not require any surgical intervention and are usually treated by conservative methods. In this case report, a pathological course of genu valgum is considered.

The exact mechanism of genu valgum in this disease is not precise. Our hypothesis is that the growth stimulation on the medial part of the femoral and tibial epiphysis and periosteums since the patient's adolescence is due to melorheostosis. Although the deformity's onset is unknown, melorheostosis is usually observed in early childhood. It usually affects one side of the body, and this disease did not cause any limb-length inequality in our patients. The deformity is controlled with a metaphyseal osteotomy after the physeal closures of the knee.

## Conclusions

Buschke-Olendorff syndrome is a rare autosomal dominant genetic disorder. It is a benign condition. This syndrome must be considered in the differential diagnosis of bone tumors in the simultaneous presence of skin and bone lesions. Furthermore, the disease may cause unilateral genu valgum in both skeletally mature and immature patients. Bone lesions are shown as melorheostosis in the plain radiographs. The treatment is symptom-based, so it must be individualized for every patient. Although this is a well-known syndrome in the relevant literature, there is a need for further studies to understand the prognosis of the disease better.
